# The Role of Exposomes in the Pathophysiology of Autoimmune Diseases I: Toxic Chemicals and Food

**DOI:** 10.3390/pathophysiology28040034

**Published:** 2021-12-18

**Authors:** Aristo Vojdani, Elroy Vojdani

**Affiliations:** 1 Immunosciences Lab, Inc., Los Angeles, CA 90035, USA; 2 Cyrex Laboratories, LLC, Phoenix, AZ 85034, USA; 3 Regenera Medical, 11620 Wilshire Blvd., Ste. 470, Los Angeles, CA 90025, USA; evojdani@gmail.com

**Keywords:** exposome, autoimmune disease, environmental factors, toxic chemicals, food, molecular mimicry

## Abstract

Autoimmune diseases affect 5–9% of the world’s population. It is now known that genetics play a relatively small part in the pathophysiology of autoimmune disorders in general, and that environmental factors have a greater role. In this review, we examine the role of the exposome, an individual’s lifetime exposure to external and internal factors, in the pathophysiology of autoimmune diseases. The most common of these environmental factors are toxic chemicals, food/diet, and infections. Toxic chemicals are in our food, drink, common products, the air, and even the land we walk on. Toxic chemicals can directly damage self-tissue and cause the release of autoantigens, or can bind to human tissue antigens and form neoantigens, which can provoke autoimmune response leading to autoimmunity. Other types of autoimmune responses can also be induced by toxic chemicals through various effects at the cellular and biochemical levels. The food we eat every day commonly has colorants, preservatives, or packaging-related chemical contamination. The food itself may be antigenic for susceptible individuals. The most common mechanism for food-related autoimmunity is molecular mimicry, in which the food’s molecular structure bears a similarity with the structure of one or more self-tissues. The solution is to detect the trigger, remove it from the environment or diet, then repair the damage to the individual’s body and health.

## 1. Introduction

Autoimmune diseases (ADs) are defined as the presence of pathogenic autoantibodies and autoantigen-reactive T-helper-1 (Th1) and Th17 cells against proven host or “self” antigens [1]. Although autoimmunity is considered a pathology of adaptive immunity, the interaction between dendritic cells (DCs), T-cell self-antigen presentation, the formation of an immunological synapse, and the identification of complexes by T cells are necessary steps for T-cell activation [2]. DCs are professional antigen-presenting cells that induce the differentiation of naïve CD4^+^ T cells into helper and effector T cells, secreting cytokines such as interleukin 12 (IL-12) and IL-23, which direct T cells to migrate to lymphoid organs or tissues, where they are primed to differentiate into Th1, Th2, Th17 or regulatory T (Treg) cells. Thus, DCs can play both immunogenic and tolerogenic roles. On the one hand, DCs have a critical role in the initiation and development of immune response and autoimmunity by promoting imbalance between Th1, Th2, Th17, and Treg cells [3,4,5,6]. On the other hand, the interaction between tolerogenic DCs and regulatory T cells (CD4^+^FoxP3^+^) plays a critical role in the induction of peripheral tolerance by the production of high levels of TGF-β and IL-10, and the prevention of the inflammatory process in Th1 and Th17 cells [7,8,9].

In autoimmune diseases, it seems that pathogenic autoreactive antibodies or autoreactive T cells against defined self-antigens appear in the blood years before the development of the active disease. In fact, it has been shown that autoantibodies can be present in the blood from 3 months to 19 years before the development of different autoimmune diseases [10,11]. This implies that autoimmune diseases are chronic; if they are not detected at the preclinical stage in order to acquire knowledge about the etiologic factors, it may not be possible to heal or find a cure for the more than 90 different autoimmune diseases that affect about 5–9% of the world’s population [12]. A recent study published in 2020 used antinuclear antibodies (ANAs), the most common biomarkers of autoimmunity, to show an alarming rise over the course of 25 years, with an overall increase of 50% [13].

## 2. Gene–Environment Interaction in Autoimmune Diseases

Currently, the more than 100 different autoimmune diseases are viewed as collections of many individual disease phenotypes that result from different gene–environment interactions that may affect both innate and adaptive immunity [14,15,16,17]. Some combinations of gene and environmental factors may lead to certain disease phenotypes; e.g., rheumatoid arthritis, thyroiditis, or systemic lupus erythematosus, whereas other combinations might not [16]. Much has been said about the influence on diseases of gene factors or the genome, but more attention needs to be paid to the role in autoimmune disorders of environmental factors.

**Figure 1:**
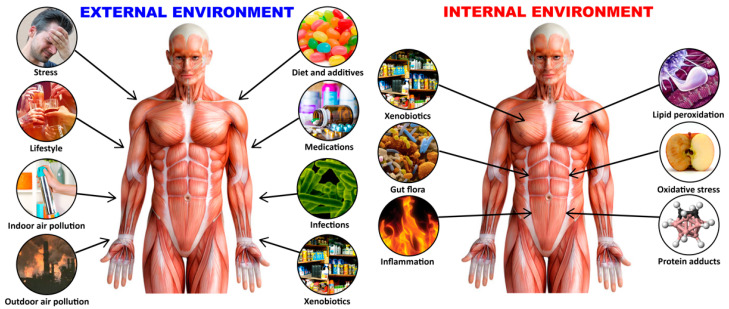
The exposome is an individual’s lifetime exposure to a variety of external and internal factors.

The exposome is considered to be the environmental equivalent of the genome, and was defined in 2005 by Wild as “life-course environmental exposures (including lifestyle factors), from the prenatal period onwards” [18]; the definition then evolved to become the systematic and comprehensive analysis of nongenetic factors influencing our health, which is essential for understanding the basis of complex disease [19,20]. Exposome today refers to lifetime exposure to a variety of external and internal sources that includes toxic chemical agents, radiation, infections, and more, from conception onwards [21,22] (see Figure 1).

As summarized by Dinse et al. [13], our genome provides the blueprints, but it is our environment writ large that determines what we become. It encompasses psychological stressors, psychosocial components such as social relations and socio-economic position, and their impact on health. This effect of genome plus exposomes on the overall health of individuals that may lead to health or diseases such as autoimmunity can be summarized as our genome + our exposome—affect → our microbiome—affect → our immunome—affect → our diseasome including autoimmunome.

Although it has been shown that environmental factors such as toxic chemicals, infections, and diet play a major role in autoimmune disorders, in the above definition of exposome, no attention has been given to undigested food components as the most prevalent modifiers of autoimmune diseases. In many studies that were conducted in our laboratory, we presented scientific evidence related to different mechanisms and associations between food proteins and peptides and their roles in autoimmune disorders [23,24,25,26,27,28,29,30].

The primary factor that controls food-related immune reactions is the oral tolerance mechanism, which can be disrupted by exposure of the host to other environmental factors, such as toxicants [13,31]. When oral tolerance breaks down, it can trigger immune reactivity against dietary antigens, which may initiate or exacerbate autoimmune disease through molecular mimicry of the food antigen with human tissue antigens [32].

In these articles, we postulated that different toxicants in food may chemically modify food proteins; if these modified food proteins cross-react with tissue antigens, the result could be autoimmune reactivity. For this reason, we feel that food components to which all humans are exposed at least three times a day or more should be added to the list of external factors of exposomes. Furthermore, because we are what our microbiome eats, the food we eat can change the commensal bacteria in our gut, and toxins can then breach the gut barrier, penetrating different organs where they can initiate an autoimmune response [33,34,35]. On the other hand, there are also foods and supplements that help maintain oral tolerance and homeostasis of the gut microbiome. A proper understanding of the link between the consumption of specific foods and autoimmunity in humans may lead to more research and greater knowledge about the possible role of a proper diet in the prevention of autoimmune diseases [32,36,37,38,39,40,41,42,43].

**Figure 2:**
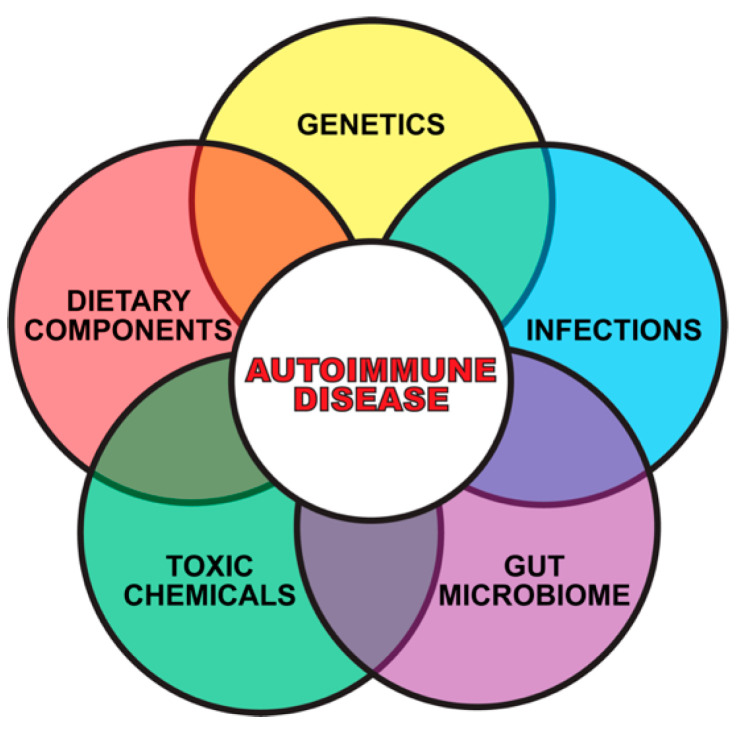
Gene plus exposome factors that contribute to autoimmune diseases.

Although accumulated evidence indicates that the immune system’s ability to distinguish between self and nonself can be negatively affected by both genetic and environmental factors, this review article focuses mainly on how the extrinsic environmental factors toxic chemicals and food antigens, two of the major components of the exposome, play a critical role in the pathophysiology of ADs (see Figure 2).

## 3. The Role of Toxic Chemicals in the Pathophysiology of Autoimmune Diseases

In recent years, many environmental agents, including synthetic chemicals, have been gaining more attention for their roles in the pathogenesis of autoimmune diseases [13,44,45,46,47,48]. However, significant gaps remain in our understanding of the cellular, molecular, and immunological mechanisms that are involved in the pathophysiology of chemical-induced autoimmunity [49,50]. About 100,000 chemicals have been approved for use in different industries, but we know very little about their effects on the immune system, and whether they may lead to autoimmunity. However, there is a lot of information about how toxic chemicals, or their metabolites can directly damage self-tissue and cause the release of autoantigens, or can bind to human tissue antigens and form neoantigens. Immune reaction against the autoantigens and neoantigens may result first in autoimmune reactivity, followed by outright autoimmune disease [13,31].

**Figure 3:**
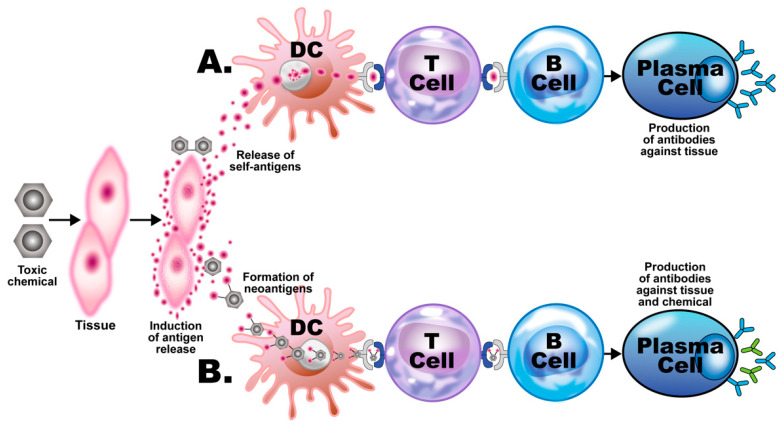
Putative mechanisms of chemical-induced autoimmunity. Toxic chemicals damage host tissue, releasing tissue self-antigens. (**A**) The self-antigens are picked up by dendritic cells (DCs) and presented to T cells, which present them to B cells, inducing them to develop into plasma cells, which produce antibodies against the host tissue. (**B**) The toxic chemical metabolites bind to the self-tissue antigens, forming neoantigens, which go through the same process of presentation and development of the B cells into plasma cells, which, in this case, produce antibodies against both the body tissue and the chemical.

**Figure 4:**
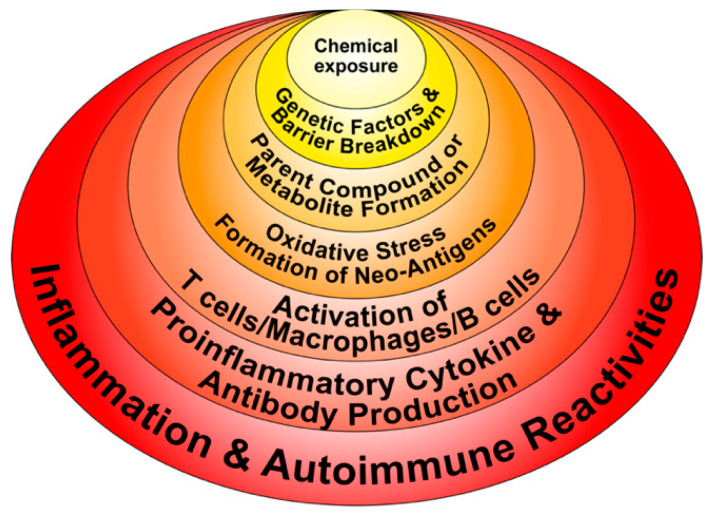
Potential molecular mechanisms implicated in chemical-induced autoimmune reactivities.

In addition to the aforementioned mechanisms of action (as shown in Figure 3 and Figure 4), these autoimmune responses and disorders can also be induced by solvents and other environmental chemicals through various effects at the cellular and biochemical levels.

1. Chemicals can alter cellular proliferation, Th1, Th2, Th3, Th17, apoptosis, and tissue-specific function;
2. Chemicals can induce protein or lipid adducts, which activate Th17 cells and induce the production of IL-17 and IL-21;
3. Chemicals can activate HSP90, inducing production of anti-HSP90 autoantibodies;
4. Chemicals can increase reactive oxygen species (ROS) production and the induction of DNA fragmentation;
5. Chemicals could interfere with iodine transportation or compete with thyroid hormones, inducing oxidative stress that leads to an inflammatory response by the thyroid gland;
6. Chemicals not only stimulate the release of ROS, but also stimulate the synthesis of nitric oxide by nitric oxide synthase;
7. Chemicals and environmental triggers in general can modify DNA methylation, inducing changes in gene expression. For example, alcohol consumption, smoking cigarettes, and exposure to environmental pollutants have been associated with autoimmunity induction through the induction of DNA methylation [51,52,53,54].

Although many environmental toxicants are implicated in the pathogenesis of ADs via different mechanisms, here we discuss the more prominent ones that are known to participate in the induction of ADs through the release of autoantigens or the formation of neoantigens.

Exposures to numerous environmental toxins have been suggested in the past as triggers for the induction of preclinical autoimmunity, but only a few have been shown to develop into full-blown autoimmune disease with a confident degree of certainty [55].

**Figure 5:**
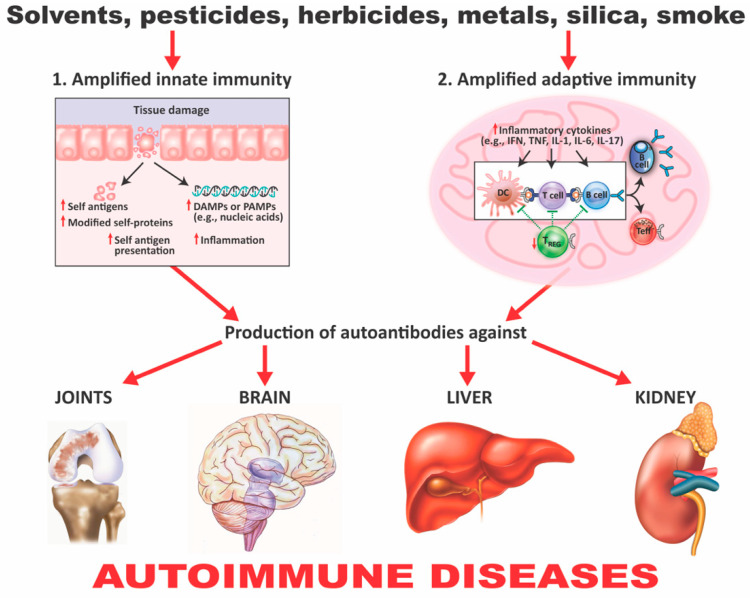
How xenobiotics can induce autoimmunity. (**1**) Amplified innate immunity. Tissue damage caused by xenobiotics can lead to the presence of cellular components and other damage-associated molecular pattern molecules (DAMPs) or pathogen-associated molecular pattern molecules (PAMPs). It can also lead to the release of self- and modified self-antigens, the presentation of these self-antigens to nontolerant lymphocytes, and the induction of inflammation. (**2**) Amplified adaptive immunity. The effects already described lead to the engagement of toll-like receptors (TLRs) and other innate sensors, the production of proinflammatory cytokines, a decrease in Treg populations, an increase in autoreactive T- and B-cell populations, and the production of autoantibodies against various self-tissues, which can contribute to autoimmune diseases. IFN = interferon; TNF = tumor necrosis factor; IL = interleukin; Treg = regulatory T cell; Teff = effector T cell.

For example, occupational exposure to crystalline silica has been linked to several autoimmune diseases, including rheumatoid arthritis (RA), systemic lupus erythematosus (SLE), systemic sclerosis (SSc), and antineutrophil cytoplasmic antibody (ANCA)-related diseases. Smoking has been identified by many studies as a great risk activity for people with RA, especially if they are positive for autoantibodies; other studies have tagged smoking as a risk for other autoimmune diseases such as SLE, multiple sclerosis (MS), and thyroid autoimmunity. A somewhat more complex association has been shown between smoking and inflammatory bowel disease, arguing that smoking contributes to Crohn’s disease [56]. Exposure to solvents or chemicals with similar structures, such as vinyl chloride, perchloroethylene, trichloroethylene (TCE), or mixed solvents has been strongly associated with SSc. Solvent exposure has also been linked by other studies with MS. Cosmetics have been associated with RA, SLE, and primary biliary cholangitis (PBC) [57,58]. In addition to their formation of neoantigens, haptenic chemicals can cause xenobiotic tissue damage, affecting both innate and adaptive immunity, which may result in autoimmune disease. This is because following this xenobiotic exposure and the subsequent tissue damage, damage-associated molecular pattern molecules (DAMPs) may be released. If the mucosal barrier is disrupted, pathogen-associated molecular pattern molecules (PAMPs) may also be released. The result is a shower of cellular debris raining down upon an assortment of molecular sensors belonging to the various cells of the immune system [59]. These sensors, including Toll-like receptors (TLRs), have vital roles in the development of immune responses against foreign antigens. Unfortunately, they also have major roles in the induction of autoimmune diseases, because they can initiate early inflammation and amplify the adaptive immune response [60,61,62]. During this process, the invading toxic chemicals form neoantigens or self-antigens that induce the production of proinflammatory cytokines such as interferon gamma (IFN-γ), tumor necrosis factor alpha (TNF-α), interleukin-1 beta (IL-1β), IL-6, and IL-17. These cytokines induce dysregulation of the regulatory T-cell population and, contrarily, the expansion of the autoreactive T- and B-cell populations, resulting first in the production of detectable autoantibodies, followed by active or full-blown autoimmunity (Figure 5).

### Mercury-Induced Autoimmunity

Mercury (Hg) has proven to be a very important factor in studying the involvement of both innate and adaptive immunity in the induction of inflammation and autoimmunity [63]. It is widely recognized as a neurotoxic metal, and, depending on the circumstances of exposure and individual susceptibility, can also act as an immunostimulant and proinflammatory agent. Exposure to mercury can occur through external pathways, such as environmental pollution, occupation, and the handling of items or products containing it; or through internal pathways, such as preservatives/adjuvants in drugs and vaccines, contaminated food, or dental amalgams. Even chronic low mercury exposure can trigger local and systemic inflammation in susceptible individuals, exacerbating the already ongoing autoimmune response in those suffering from autoimmunity. Exposure to Hg can cause dysregulation of autoimmune responses and aggravation of the immunotoxic effects associated with elevated titers of autoantibodies detected in serum [64], as shown below:

1. Mercury-induced proliferation of human lymphocytes has been shown to occur 6 days postexposure with increased expression of several cytokines, including TNF-α, IL-1β, IL-6, and IL-8 in peripheral blood mononuclear cells (PBMCs). This lymphoproliferative response drives Th2 cell response [65];
2. Very low (micromolar) concentration of mercuric chloride (HgCl_2_) can negatively affect the function of neutrophils; this is demonstrated by the enhanced production of hydrogen peroxide (H_2_O_2_), increased lysosomal enzymes, and the formation of neutrophil extracellular traps. These findings indicate the involvement of these cells in local tissue injury induced by mercury [66];
3. In epidemiological studies, elevated levels of the inflammatory markers IFN-γ, TNF-α, and IL-1β were found in the sera of Amazonian gold miners in Brazil. Mercury was used to recover minute pieces of gold. Fish consumers from the same place who were exposed to mercury also showed increased levels of IFN-γ, IL-4, IL-6, and IL-17 cytokines [67];
4. Mercury exposure is associated with the production of autoantibodies. In the same epidemiological study described above [67], autoantibodies were detected in the artisanal Amazonian gold miners. A positive correlation also was shown between the consumption of fish by the Amazonians and the presence of antinuclear antibodies (ANA) [67,68]. This same positive correlation was shown between fish consumption by members of the Cheyenne River Sioux tribe of the female gender and the presence of ANA in their blood [69]. Mercury was also detected in the blood of Faroese children and associated with multiple neural and non-neural IgM antibodies.

The association between mercury levels and autoantibodies has also been investigated by the National Health and Nutrition Examination Survey (NHANES) in different cohorts. NHANES is a program that assesses the health and nutritional status of the United States population. The 2007–2008 NHANES survey found an association between thyroglobulin autoantibody positivity and the upper quintile of blood mercury (>1.81 μg/L) in women 20 years and older [70], while the 1999–2004 survey found an association between ANA in women 16–49 years old and mercury levels in their hair and blood [71]. Overall, the generation of IgG ANA, antinucleolar autoantibodies (ANoA), dsDNA antibodies, and immune complex deposits in glomeruli, blood vessels, and skin may result in the nephrotic syndrome observed in patients with lupus.

5. Mercury-induced nephrotic syndrome is an established outcome of mercury exposure in humans [72] through such things as mercury-containing cosmetics, hair dyes, mercury-containing pills, and occupational contact [73,74,75]. A review of the literature found that out of 26 renal biopsy cases, 21 had glomerular diseases, with the major pathological observations being membranous glomerulonephritis (15 patients) and minimal change disease (4 patients). Immune complexes and autoantibodies have been found in some patients, but not in others [72,73,74,75,76]. The mechanisms that lead to mercury-induced glomerular injury in humans remain to be definitively identified. It is known that mercury shows significant renal tubular toxicity, and it is possible that this induces the release of self-antigens and resulting cytokine-associated inflammatory response [77,78];
6. In animal studies of mercury-induced autoimmunity, it has been shown that mercury exposure can clearly induce systemic autoimmunity in different animal species. This gives support to the biological plausibility of mercury as a factor in autoimmune diseases in humans. Many studies, mostly with mice, have demonstrated that mercury-induced autoimmunity can result from different modes of exposure, including oral ingestion of HgCl_2_, inhalation of mercury vapor, dental or periodontal implants containing dental amalgam, or subcutaneous injection [62,79,80,81,82,83].
7. Mercury-induced cell death can lead to the production of antibodies against the destroyed cell’s components. Antiglomerular basement membrane (GBM) autoantibodies have been reported in rats and rabbits; ANA has been found in rats. Mercury-induced ANA in mice was found to include ANoA, which has now been identified as an antibody against fibrillarin, a protein component of box C/D small nucleolar ribonucleoproteins (snoRNPs) particles, the main functions of which are methylation and the processing of pre-rRNA [84,85,86,87,88,89].

Unlike HgCl_2_-induced responses to other nuclear antigens, this antifibrillarin response is MHC class II-restricted. It has similarities to the antifibrillarin response in human autoimmunity, such as the recognition of an epitope conserved from humans to yeast. This modification negatively affects both human and mouse antifibrillarin binding, indicating that unmodified native fibrillarin is the dominant B-cell antigen [90,91].

This also indicates that mercury-induced cell death results in proteolytic cleavage of fibrillarin to a 19 kDa fragment. This fragment is capable of inducing antifibrillarin autoantibodies. Together, all of this suggests that mercury-induced cell death can generate novel protein fragments that can become antigenic determinants for self-reactive T lymphocytes [92,93].

**Figure 6:**
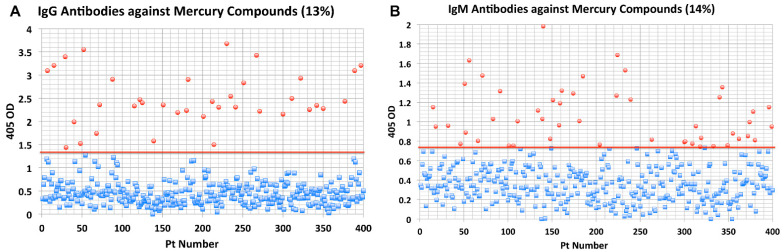
Results for mercury antibodies expressed as optical density (OD) at 405 nm in the form of scattergrams: (**A**) mercury immunoglobulin G (IgG); (**B**) mercury immunoglobulin M (IgM).

8. Elevated levels of antibodies against xenobiotics (including mercury) have been found in a subgroup of healthy subjects. By acting as a hapten, mercury can bind to a high-molecular-weight carrier protein, such as human serum albumin (HSA), causing the immune system to mistakenly “recognize” self-tissue as an invader and launch an immune response against it, leading to autoimmunity. In one of our studies [30], we measured IgG and IgM antibodies against mercury and 11 other chemicals bound to HSA in the blood of supposedly healthy donors using ELISA methodology. We found that 13% (IgG) and 14% (IgM) of tested individuals showed significant antibody elevation against mercury (see Figure 6). The percentage of elevation against the other 11 chemicals ranged from 8% to 22% for IgG, and 13% to 18% for IgM.

**Figure 7:**
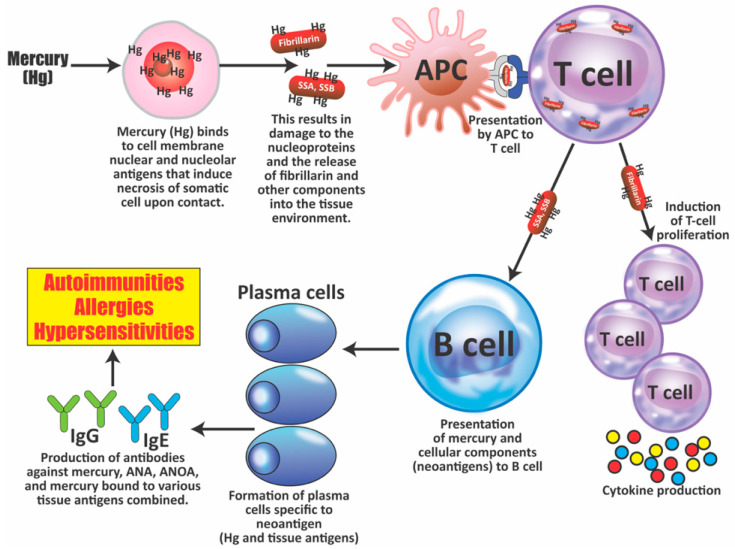
The pathophysiology of inflammatory and autoimmune responses induced by mercury exposure. Within minutes to hours at the site of injection, mercury (Hg) binds to cellular components and then induces necrosis of somatic cells; this results not only in cell death, but also in tissue damage, the release of lysosomal enzymes, and the proteolysis of self-proteins such as nuclear and nucleolar proteins, which then carry mercury, helping in the formation of neoantigens. These antigens or peptides are taken up by antigen-presenting cells (APCs) and then presented first to T cells, and then to B cells. Mercury exposure also induces the production of B-cell activating factors by APCs, leading to the proliferation of both T cells and B cells. Mercury also promotes the production of cytokines, such as IL-4, and a Th2 response, inducing B cells to become plasma cells, which produce immunoglobulin G (IgG) or immunoglobulin E (IgE) antibodies against mercury, nuclear and nucleolar antigens such as fibrillarin, and chromatin and other autoantigens. The binding of these antibodies to antigens after activation of the complement cascade and binding to C1Q may result in the deposition of immune complexes in the kidney and possibly the joints. SSA = Sjögren’s syndrome A; SSB = Sjögren’s syndrome B; ANA = antinuclear antibodies; ANOA = antinucleolar antibodies.

This detection of antibodies against a variety of protein adducts may indicate chronic exposure to these chemical haptens in about 20% of the tested individuals. Protein adduct formation could be one of the mechanisms by which environmental chemicals induce autoimmune reactivity in a significant percentage of the population. The postulated pathophysiology of inflammatory and autoimmune responses induced by mercury that may result in antibody production against mercury and different autoantigens is shown in Figure 7.

## 4. Food Coloring and Autoimmunity

As discussed in the previous section, animal and human exposure to mercury in its different forms was shown to be associated with inflammatory and autoimmune reactivity; however, relatively little is known or has been discussed regarding the role of food coloring in the pathophysiology of autoimmune disorders. In fact, using Med Search, we found only two different articles: one by this author under the title “Immune reactivity to food coloring” [94], and a second article by Lerner and Matthias, who described how industrial food additives contribute to the rising incidence of autoimmune diseases [33]. Due to this lack of information on food coloring’s possible involvement in inflammation and autoimmunity, we decided to investigate the matter in this current article.

Why is food coloring necessary? Although fresh food in its natural state is usually visually appealing, processed food generally tends to lose its luster, and as a result the food industry enhances the appearance of food with food coloring. Artificial food dyes are synthesized from compounds found in petroleum, and have been approved by the US Food and Drug Administration (FDA). These colorants are universally used not only for food, but also by the pharmaceutical industry to make their products at least more visually palatable. There are some natural food colorants, but artificial dyes can be produced in bright hues not possible with natural colorants, and have the additional advantages of being easier to obtain, longer lasting, and, as a final argument, cheaper. It is most likely for these reasons that in the last 50 years, the use of artificial coloring used in foods has increased by 500%. Unfortunately, this increase has been accompanied by increasing reports of allergic and other immune reactive disorders, as well as a disturbing rise in behavioral problems in children, such as aggression, attention deficit disorder (ADD) [95], and attention-deficit/hyperactivity disorder (ADHD) [96]. The greatest foreign antigenic challenge that the immune system faces is through the ingestion of food. This is not surprising, considering that most people eat at least three times a day, in a process that involves putting things into the body that are generally substances foreign to it. Artificial colors do not just infiltrate the body through food. They can also be absorbed through the skin via cosmetic and pharmaceutical products. Since the molecules of artificial colorants are small, it is very difficult for the immune system to defend the body against them. The molecules are also able to bind not just to food proteins, but to the body’s own proteins as well. This ability to bind to an individual’s own proteins means two things: that the artificial food molecule will basically disguise itself because the immune system will mistakenly think it as a self-protein; or that the immune system does recognize that the colorant molecule bound to the body protein is a foreign antigen, but then mistakenly also identifies the body tissue bound to the color molecule as a foreign antigen, and produces an immune response against both the actual foreign antigen and the self-protein, with significant immunological consequences. This can result in activation of the inflammatory cascade, induction of intestinal permeability to large antigenic molecules, cross-reactivities, autoimmunity, and even neurobehavioral disorders. In the past decade, a 47% increase in diagnoses of ADHD in high-school boys was found by the Centers for Disease Control (CDC). This is still not as alarming as the legal number of synthetic colorants permitted by the FDA to be mixed into or applied to the foods, pharmaceuticals, and cosmetic products that we actually ingest or otherwise use on a regular basis. Sadly, the general public is for the most part oblivious to the shockingly dangerous true nature behind the enticing bright colors of synthetic food dyes [94].

### 4.1. The Binding of Food Colors to Human Tissue Proteins Contributes to the Pathophysiology of Autoimmunity

Significant amounts of coloring food additives such as Allura Red, erythrosine, Brilliant Blue, Patent Blue V, and tartrazine can enter the bloodstream through the GI tract by the ingestion of food or orally taken medications, or via skin absorption through the application of cosmetics, skin creams, shaving creams, and other skin-contact products [97,98].

**Figure 8:**
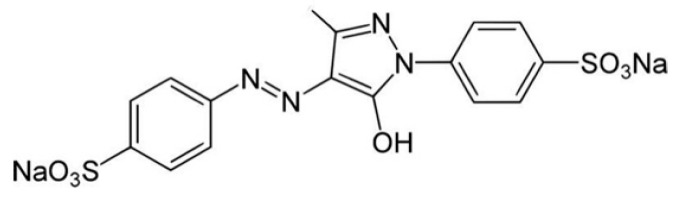
Molecular formula for Tartrazine: C_18_H_14_N_2_Na_2_O_8_S_2_ (molecular weight 534.3).

Because most food additives, such as tartrazine (see Figure 8), carry very active chemical groups, their penetration into the body can result in their interaction with human proteins and the formation of neoantigens. The following foods may contain Tartrazine: hard candy, cotton candy, gummy bears, marshmallow treats, and other confectionary products; gelatins and instant puddings; cake mixes, processed pastries, biscuits, and cookies; and many more [99]. This is just one example of a food colorant that can react with human tissue components due to its chemical structure.

### 4.2. The Effects of Food Coloring on Protein Digestibility

**Figure 9:**
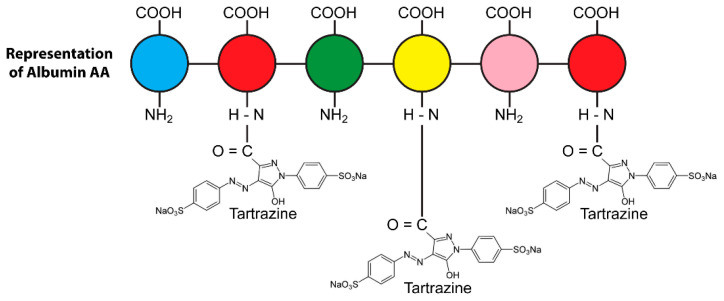
Covalent binding of tartrazine through carboxylic group to human serum albumin amino groups, forming tartrazine–protein adduct.

One of the reasons artificial food colors are so popular around the world is that they form stable complexes with proteins, thus giving uniform color distribution to all common foods [100,101]. This is because food colors are generally ionic in nature, and so, they can interact strongly with proteins to form covalent bonds [102]. Unfortunately, this covalent binding of colors to human proteins is also a major mechanism for the induction of the immune reactivity and hypersensitivity associated with many synthetic food colors [103]. Studies have shown that food coloring binds covalently to different host tissue proteins, such as hemoglobin and human serum albumin (HSA) [104,105,106,107]. The different food colors (Allura Red, tartrazine, Brilliant Blue, etc.) bind with proteins in various food environments, forming food protein–color complexes. In the digestive system, these complexes are supposed to be digested by proteolytic enzymes, but because the different color additives bind to the active sites of the food proteins, the binding can affect the tryptic digestibility of the different proteins [104]. This binding of food colors to food proteins, forming stable food protein–color complexes, is shown in Figure 9.

**Figure 10:**
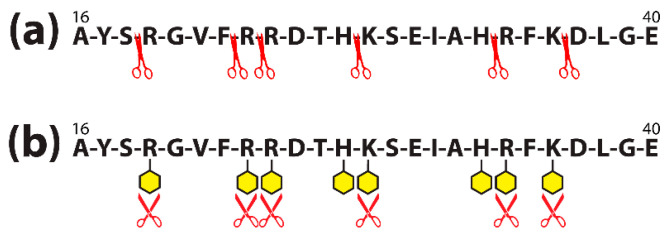
Amino acid sequence of albumin peptide before (**a**) and after (**b**) food colorant binds to different amino acids contained in the chain. (**a**) Trypsin (symbolized by red scissors) is shown cleaving the amino acid chain of a 16–40 sequence of albumin; (**b**) colorants (symbolized by yellow hexagons) bind to the major amino acids arginine (R), histidine (H), and lysine (K) present in the albumin sequence, making it difficult for the trypsin to cleave the sequence, and decreasing digestive effectivity.

Proteins are made of many amino acids (AAs); each AA contains both amino (NH2) and carboxylic (COOH) groups. For their part, almost all food colors carry very active groups. The AA amino and carboxylic groups are perfect for binding with the food additives’ active groups to form covalent bonds. Figure 10a shows how a food protein sequence would normally be cut apart by the enzyme trypsin (symbolized by scissors) during digestion. Figure 10b shows how the binding of the active additive groups (symbolized by yellow hexagons) to the lysine (K), arginine (R), and histidine (H) sites of the food protein sequence forms covalent complexes that block the trypsin enzymes from cleaving the proteins, significantly inhibiting the ability of the enzyme to digest the food [104].

One study [101] showed that 156 out of 607 amino acids of bovine serum albumin (BSA) bound with the dyes. The binding interfered with trypsin cleavage as described above and shown in Figure 10. If this binding occurs in the digestive tract, it can result in an accumulation of undigested food proteins or immunogenic peptides in the gut, which can trigger an inflammatory cascade.

The study in [104] concluded that due to this binding, the consumption of food colors, especially artificial color additives, that is prevalent in the modern lifestyle has significant immunological consequences, resulting in increased intestinal permeability to large antigenic molecules [108]. This could lead to cross-reactivity and autoimmunity [25,109]; if the colorants or their metabolites form color–protein neoantigens throughout the body, the immune system could respond with autoimmunity against the self-protein with which the colorant has bonded. For instance, if colors bind to hemoglobin, then the immune system may attack the body’s own red blood cells (RBCs), resulting in a low RBC count, and liver autoimmunity can arise from colors binding to liver enzymes.

This was confirmed by another study [104], which showed the coloring tartrazine forming a complex with hemoglobin, with toxic results. Similarly, another study [110] showed that the binding of food dye to HSA induced conformational changes in the molecular structure of the HSA, resulting in the production of autoantibodies.

The measurement of the IgG antibody to food coloring bound to HSA is routinely performed in our own laboratory [32]. After examining the elevation in IgG antibodies against these neoantigens, we found that from 12–15% of the tested blood specimens had significantly higher levels of food coloring HSA antibodies at 2SD above the mean.

Despite all these findings and numerous articles published in scientific journals, scientists, to a great extent, are unfortunately still unaware of the perils of the widespread use of food color additives today.

The corresponding author himself fell prey to the lures of artificial coloring. One of his favorite types was Indian cuisine, particularly Tandoori chicken. He enjoyed eating it for many years, delighting in its taste, aroma, and color, thinking that the dish’s bright red color was from natural spices. He was shocked when he finally learned that his favorite food’s signature crimson color came, not from paprika and cayenne pepper, as he had believed, but from artificial food coloring, which is the only thing that can bind covalently to a meat protein and give it such a bright red hue.

The author is now aware that food coloring can cause the following issues:

1. Breakdown in oral tolerance;
2. Decreased efficiency of digestive enzymes;
3. Increase in intestinal permeability;
4. Liver toxicity;
5. Mitochondrial dysfunction;
6. Hypersensitivity;
7. Food immune reactivity;
8. Asthma, allergic rhinitis, angioedema;
9. Atopic dermatitis;
10. Interference with neurotransmission;
11. Neurobehavioral disorders;
12. Reproductive abnormalities.

With this knowledge, the author enjoins everyone not to be deceived by the visual enticements of brightly colored candies, pastries, drinks, medications, cosmetics, and—yes—even Tandoori chicken [94].

Mercury and food coloring are only two examples of the many toxic chemicals we encounter in everyday life that, through various mechanisms, contribute to the pathophysiology of autoimmune disease. Toxic chemicals can be present in the air we breathe, the water we drink, the land we walk on, as preservatives in our food, as additives in our medicines and even vaccines, as insecticides on our pretty flowers and nutritious vegetables, and as packaging and integral parts of the many products we use daily. Unfortunately, the modern techno-industrial lifestyle that we have adopted over the years encourages us to ignore the dangers posed to our health by these poisons in the name of convenience and instant gratification.

## 5. Reaction of the Immune System to Food Antigens and Its Contribution to Autoimmune Disorders

The oral tolerance mechanism is the primary factor that controls food-triggered immune reactivity. A breakdown or failure in oral tolerance activates immune reactions against dietary antigens; if these food antigens share homology with human tissue antigens, this may initiate or exacerbate autoimmune disorders [32]. In the past two decades, there has been great progress in the search for food antigen peptides that have a significant similarity with autoantigens that have been associated with autoimmune diseases [111,112,113,114,115,116]. Normally, due to oral tolerance, foods are digested without triggering the immune system, otherwise we would all die of starvation or anaphylactic shock. However, undigested food proteins that manage to get into places where they do not belong can activate the body’s immune defenses. The immune system can then mistakenly classify the errant food protein as an invader and register the food’s epitope—the part of the antigen that is recognized by the immune system. Unfortunately, if this specific food peptide epitope shares a significant commonality or similarity with the body’s own tissues, it can elicit a T- or B-cell immune response that can lead to different diseases, such as food allergy, psoriatic arthritis, chronic idiopathic urticaria, and even autoimmune disorders [111,112,113]. In such an autoimmune response, T-cell clones specific to particular food antigen epitopes may be generated in the gut mucosa and then be transported to particular sites; for example, the joints, where they proliferate due to the peptide homology between the food antigen epitopes and the joint tissue epitopes. The resulting local inflammation, upregulation of major histocompatibility complex molecules, release of additional self-antigens, and/or epitope spreading can lead to a repeating, self-perpetuating process of organ inflammation and destruction, culminating in autoimmunity [112]. Despite the great variety of food antigens available for consumption in today’s world, the pathological mechanism described above has only been extensively investigated for only a relative few food proteins and peptides, some of which are discussed in the following section [117,118,119].

### 5.1. Autoimmunity, Wheat, and Milk

In the past decades, the discussion has increased regarding the connection between various health disorders and food immune reactivity, especially reactivity to wheat and milk [117,118,119].

Many researchers have identified several gluten peptides with the capacity to stimulate intestinal T-helper cells in patients with celiac disease (CD) [120,121,122,123,124,125,126]. The studies have demonstrated that intestinal T cells from CD patients reacted to a broad variety of peptides. One study [123] isolated T cells from CD patients and screened them for 21 different peptides, ranging from α-, γ-, and ω-gliadins to glutenins. In other studies [124,125], many peptides from the α-gliadin family were recognized in some patients, whereas only one peptide caused lymphocyte stimulation and interferon production in other patients. Additionally, one of the corresponding author’s own studies [126] showed that patients with Crohn’s disease and nonceliac gluten sensitivity (NCGS) reacted to and produced IgG and IgA antibodies against a repertoire of wheat antigens that included α-, γ-, and ω-gliadins; glutenins; gluteomorphins; and wheat germ agglutinins (WGAs). Chronic exposure to environmental factors such as wheat can not only cause NCGS and CD, but also lead to inflammation and even autoimmunity if not treated in time [127,128,129]. In fact, in another of the corresponding author’s articles, he showed that monoclonal and polyclonal antibodies made against only α-gliadin 33-mer were still enough to result in antibody reactivity against hepatocyte cytochrome P450, collagen, asialoganglioside, glutamic acid decarboxylase (GAD), myelin basic protein (MBP), cerebellar, and synapsin [25].

In another study, it was shown that gliadin peptides carrying the QQQPFP epitope interacted directly with actin or smooth muscle, resulting in rearrangement of the actin cytoskeleton and possible autoimmunity against actin and the gliadin peptide [130]. Most CD patients have circulating antibodies against wheat proteins, and these antibodies can react against bone structures, which may be one of the reasons why osteopenia and osteoporosis are well-known complications of NCGS and CD. The immunoreactivity detected in NCGS and CD sera may be due in part to bone transglutaminase (tTG) and other bone antigens acting as autoantigens [131].

Evidence also links CD with heart disease, such as autoimmune myocarditis. A study involving patients with autoimmune myocarditis detected an autoimmune process against cardiac antigens that could play an important part in the pathogenesis of inflammatory heart disease. This was supported by the discovery that the removal of tTG and antiendomysial antibodies from the patients’ sera was accompanied by an improvement of cardiac function and ventricular arrhythmias [132]. In some patients, gluten sensitivity presents as dermatitis herpetiformis (DH) instead of as CD. In these patients, the target autoantigen appears to be epidermal tTG-3 [133,134].

CD and NCGS have also been implicated in a variety of neurological disorders. In gluten ataxia, one of the most common brain-related manifestations of gluten sensitivity, the antibodies that are released when digesting gluten attack part of the brain by mistake. The known biomarkers for this disease are IgG and IgA antibodies to gliadin, tTG-6, cerebellar, and GAD-65. The symptoms of gluten ataxia have been shown to improve on a gluten-free diet [135,136].

About 25% of CD patients also have gluten or peripheral neuropathy, which can be triggered by the gluten antibody [128]. The biomarkers commonly detected in these patients are antibodies to gliadin, tTG and asialoganglioside [137,138,139,140].

In patients with multiple sclerosis (MS), some studies have shown an increase in the prevalence of antibodies to gliadin and myelin basic protein (MBP) [24,141].

Some patients with gluten sensitivity have also been confirmed with neuromyelitis optica (NMO), a rare central nervous system disorder in which the immune system attacks the spinal cord and optic nerves, potentially leading to permanent blindness and even paralysis. In NMO patients, antibodies have been detected against neural antigens, particularly against aquaporin 4 (AQP4) [142].

**Figure 11:**
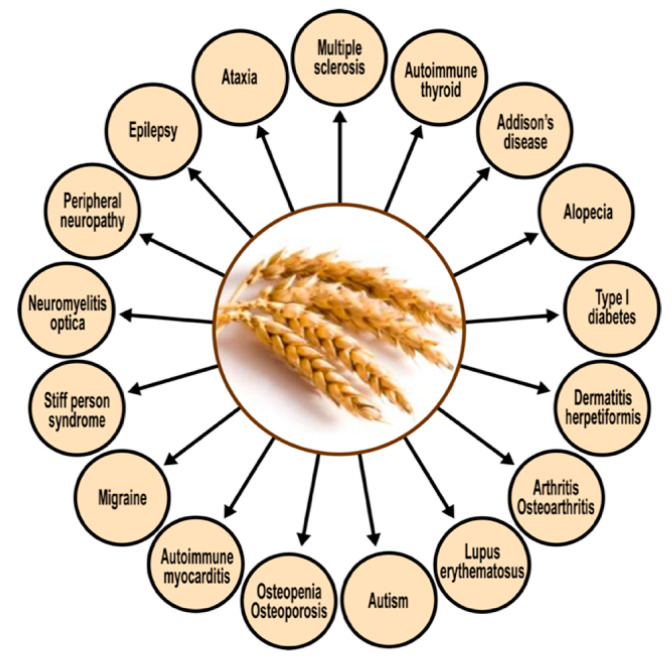
Spectrum of autoimmune disorders associated with wheat proteomes.

The corresponding author’s own studies [23,24] have shown a significant degree of cross-reactivity between gliadin and at least five different neuronal antigens (MBP, asialoganglioside, synapsin, GAD-65, and cerebellar). It should thus be not so surprising that so many autoimmune reactivities linked to gluten sensitivity target the nervous system and related tissues. Figure 11 shows the spectrum of autoimmunity associated with protein components of wheat. The cross-reactivity between gliadin antibodies and a range of human self-tissue antigens may explain how immune reactivity in a target site distant from the digestive system could still be due to shared homology or common epitopes [139].

The above figure shows that CD and NCGS present extraintestinal symptomatology in almost every organ of the body, especially in the brain [139]. Therefore, the detection of antibodies against wheat proteomes, and their possible cross-reaction with various human tissue antigens, could be vital for the early detection of gluten/gliadin-related autoimmune reactivities.

Based on the antibodies detected, the removal of immunological triggers (in this case, the activating food antigens) should be further investigated as possible treatment modalities for all the manifestations shown in Figure 11 [139].

### 5.2. Immune Reactivity, Autoimmunity, and Milk Proteins

**Figure 12:**
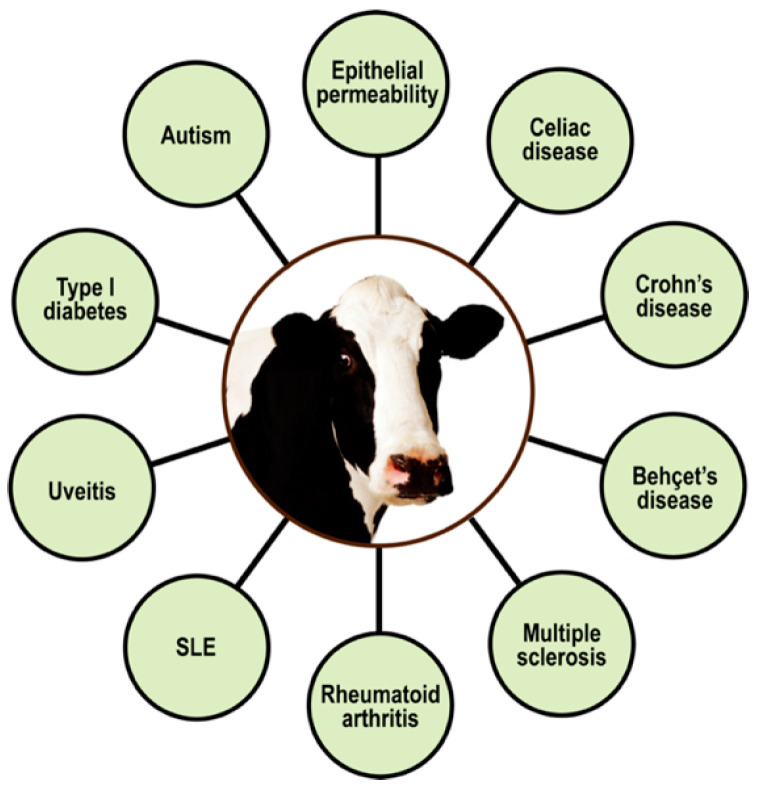
Spectrum of autoimmune disorders associated with milk proteomes. SLE = systemic lupus erythematosus.

Since ages past, people have generally believed that milk is one of the most important parts of a healthy diet, saying that it is good for you, it strengthens your bones, mother’s milk, etc. Unfortunately, modern science has now discovered that cow’s milk proteins are the most common food allergens affecting young infants, children, and adults alike [143,144]. Of these allergenic milk proteins, the major ones are α-casein, β-casein, κ-casein, and β-lactoglobulin. Awareness is increasing that the problem is not just IgE-mediated allergic reactions to cow’s milk and its proteins, but that the early consumption of milk, basically a foreign liquid secretion from a completely different species, may also increase the risks of developing autoimmune diseases (e.g., Behçet’s disease, CD, Crohn’s disease, lupus, MS, type 1 diabetes, uveitis, etc.), as shown in Figure 12 [145,146,147,148,149,150,151,152,153,154,155].

The findings of these studies [24,141,142,143,144,145,146,147,148,149,150,151,152,153,154,155] were supported by the detection of much higher levels of IgG and IgA antibodies against milk proteins in individuals suffering from these diseases, in comparison to normal or healthy controls. The conclusion of these studies was that the active immune responses against cow’s milk proteomes play an important role in the pathogenesis of autoimmune diseases [146,147,148,149,150,151,152,153,154,155,156,157,158].

**Figure 13:**
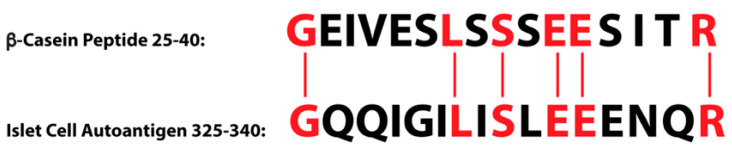
Antigenic similarity between cow’s milk protein β-casein and islet cell autoantigen.

For instance, in type 1 diabetes, significant evidence has been gathered regarding the association between the consumption of cow’s milk and the disease [147,148,149,150]. The consumption of milk by certain susceptible individuals may prompt an immune reaction or response to milk proteins. Thus primed, the immune system may then mistakenly recognize islet cell antigens that possess peptide sequence similarity with those milk proteins, and then react against them. The homology between the islet cell and β-casein peptide sequences is shown in Figure 13.

Based on this sequence homology between cow’s milk protein and β-cell components of islet cells, some researchers have concluded that type 1 diabetes may be caused by autoreactive CD4^+^ Th1 lymphocyte clones, citing the reactivity of β-casein-specific T cells with β-cell antigens of islet cells [157,158].

**Figure 14:**
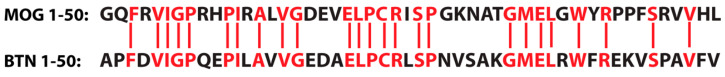
Similarity between cow’s milk protein BTN and human MOG. Only one out of many cross-reactive peptides is shown. MOG = myelin oligodendrocyte glycoprotein; BTN = butyrophilin.

Multiple sclerosis (MS) is another autoimmune disease that has been associated with milk proteins. Most neuroautoimmune disorders such as MS are believed to be inflammatory disorders in which diet can play a significant role in the induction of autoimmunity [159,160,161,162,163,164,165,166,167,168,169,170,171,172,173,174,175,176,177,178,179,180,181,182,183,184,185,186,187,188,189,190,191,192]. A comparison between MS mortality rates from 1949–1967 and food consumption data from the United States found an extremely high correlation between mortality and milk consumption, and an inverse relation between mortality and the consumption of fish and vegetable fat [163]. In seeking an explanation for this correlation between milk consumption and MS mortality, studies [164,165,166] found the highest levels of sequence homology between the major milk fat protein butyrophilin (BTN) and myelin oligodendrocyte glycoprotein (MOG), a protein important in the myelination or sheathing process of the nerves in the central nervous system. This MOG is a major target for the autoimmune reactivity generated in MS experimental autoimmune encephalomyelitis (EAE), the animal model of MS [151]. In animals with EAE, MOG is the only myelin autoantigen known to induce both an encephalitogenic CD4^+^ T-cell response and a demyelinating autoantibody response [167]. The CD4^+^ response disrupts the blood–brain barrier (BBB), while the MOG autoantibodies bind to the MOG exposed on the myelin surface and mediate demyelination [168]. In another study [150], a sequence of 120 amino acids in the MOG peptide was compared to milk BTN, showing 50% similarity between nine different peptides of MOG and BTN, one of which is shown in Figure 14.

Since we can see that homology between some milk proteins and some human proteins have the possibility of leading to immune reactivity and even outright autoimmune disease, the consumption of milk products by individuals with neuroautoimmune disorders is obviously contraindicated, or should at the least be scrutinized and monitored, especially for patients who show high levels of antibodies against MOG and other neural antigens.

### 5.3. Neuroautoimmunity Due to Food Containing Aquaporins

Aquaporins are integral membrane proteins that act as water channels to conduct water through the cell membrane. Aquaporin-4 (AQP4) is the most common aquaporin in the brain, spinal cord, and optic nerve. It is found in endothelial cells, ependymocytes, and astrocyte foot processes at the BBB, and in the epithelial cells of many organs [169].

**Figure 15:**
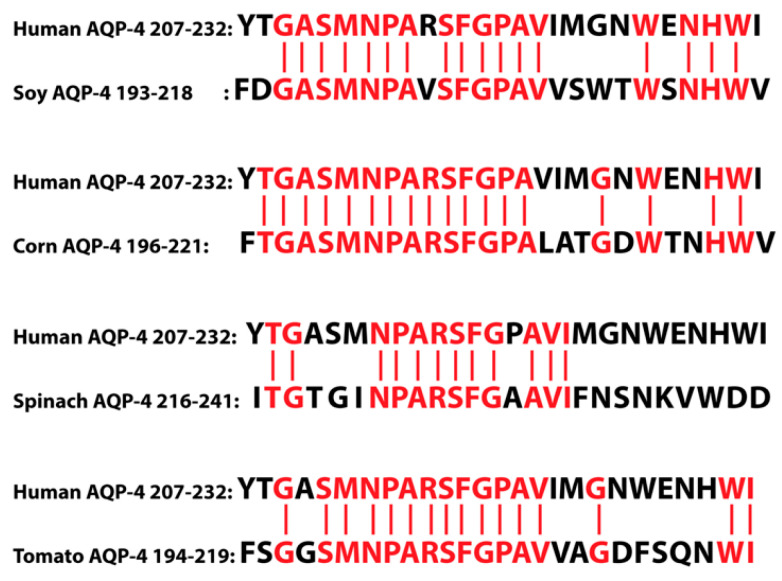
Similarity between different plant AQP4s and human AQP4. AQP = aquaporin.

Aquaporins are also found in plant food sources, and they become highly stable after undergoing food preparation. This means that they may make it through the process of digestion as more or less intact proteins or peptides, and if the body’s immune tolerance fails, these aquaporin molecules may become antigenic, resulting in an antiaquaporin immune response [170,171]. The problem is that plant food aquaporin shows similarity with human aquaporin, as shown in Figure 15.

As shown in Figure 15, AQP4 from foods such as soy, corn, spinach, and tomato share sequence homology with human AQP4. Human AQP4 also shares homology with serpins, which are legume serine proteinase inhibitors found in peas, beans, lentils, peanuts, lupin, clover, and alfalfa [170,171]. The human aquaporin can thus cross-react with these food aquaporins and serpins, resulting in immune reactivity and the production of autoantibodies against not only the food aquaporins, but the host’s own aquaporin channels as well. This can have dire consequences if these autoantibodies manage to cross the BBB, because, as we mentioned earlier, AQP4 is the most common aquaporin in the brain, spinal cord, and optic nerve, and the autoantibodies that manage to cross over can now attack the human aquaporins that are so prevalent and so necessary for the functioning of those areas. For example, neuromyelitis optica (NMO), or Devic’s disease, is a severe neuroautoimmune inflammatory disorder. A great majority of NMO cases are caused by IgG1 autoantibodies binding to the host’s AQP4 in critical neural areas, resulting in complications that can range from muscle weakness and loss of bladder/bowel control to paralysis and blindness [142,170,172].

**Figure 16:**
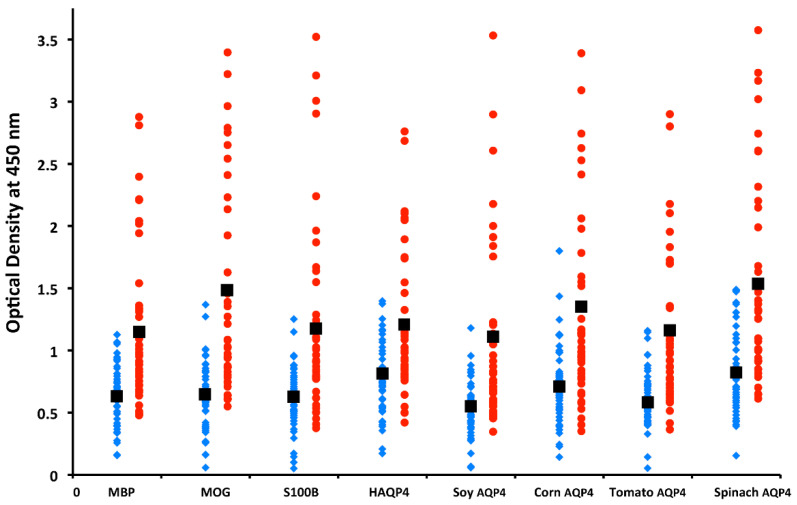
IgM antibody values of MS patients (red circles) versus controls (blue diamonds) for myelin basic protein (MBP), myelin oligodendrocyte glycoprotein (MOG), S100B, human aquaporin 4 (AQP4), and AQP4 for soy, corn, tomato, and spinach were significantly higher for patients than controls. IgG and IgA results showed similar differences between patients and controls.

In our own lab, we measured antibodies against plant and human aquaporins in blood samples from patients with multiple sclerosis, and found significant elevations in antibodies against different AQP4 plant peptides and neuronal antigens such as MBP, MOG, and S100B [173], as shown in Figure 16. These results supported the involvement of food source aquaporins in neuroimmune disorders, and this information may help in the development of dietary modifications for patients with NMO, MS, and other autoimmune disorders of the nervous system.

### 5.4. Cross-Reactivity and Sequence Homology between Food Products and Alpha-Synuclein

Parkinson’s disease is characterized by the abnormal folding of alpha-synuclein (aSN), a neuronal protein that is abundant in the brain and is found in smaller amounts in the heart, muscle, and other tissues. It regulates synaptic vesicle trafficking and subsequent neurotransmitter release, and is localized in the substantia nigra in the form of Lewy bodies. In the same way that some foods share sequence homology with human AQP4, many foods, including plants such as soy, beans, peanuts, tomato, and wheat, as well as crustaceans such as shrimp, contain different proteins that also share peptide similarity with aSN [174,175,176,177,178,179]. These aSN sequences are also highly conserved in vertebrates, especially mammals, which is why food products from animals, particularly mammals, are the main sources of aSN that reach the gut [180,181,182,183,184,185]. It has been hypothesized that foods and other environmental factors can reach the peripheral nervous system through the gastrointestinal system or nasal cavity, then finally reach the brain, causing the aSN to misfold [186,187,188].

Based on the above studies, in our own recent article [189], we hypothesized that luminal food peptides that share cross-reactive epitopes with human aSN and share sequence homology with human brain antigens are involved in synucleinopathies such as Parkinson’s. We used the Basic Local Alignment Search Tool sequence matching program to attempt to find matches between aSN and different foods. Even with a percentage of identity cutoff set at 50%, the resulting number of matches was overwhelming, with from 4 to more than 20 peptide sequence matches with aSN. The foods with the greatest number of matches were yeast, soybean agglutinin, latex hevein, wheat germ agglutinin (WGA), peanut, pea lectin, potato, bean agglutinin, and shrimp [189]. Furthermore, when monoclonal antibodies made against recombinant aSN protein were applied to the antigens of 180 frequently consumed food products, these antibodies had moderate to strong reactions with 86 out of the 180 food antigens. We concluded that the aSN antibody’s cross-reactivity with common foods due to the molecular mimicry or homology between specific peptide sequences reinforces an autoimmune explanation for the neurodegeneration characteristic of Parkinson’s disease [189].

### 5.5. Contribution of Lectins and Agglutinins to Autoimmune Diseases

Lectins are carbohydrate-binding proteins present throughout nature. They can cause the agglutination of particular cells, or the precipitation of glycoconjugates and polysaccharides. They are highly specific for sugar groups that are part of other molecules, and have a role in biological recognition phenomena involving cells, carbohydrates, and proteins [190,191]. When consumed in excess by individuals with dysfunctional enzymes, lectins can disrupt digestion, cause nutrient deficiencies, and even cause severe intestinal damage due to their binding properties. This can lead to the disruption of intestinal barrier integrity, which is the gateway to autoimmunity. Once again, cross-reactivity may play a role in the pathogenesis of autoimmunity, in this case due to shared amino acid peptides or sequences between dietary lectins and various body tissues, resulting in the production of antibodies against both the lectins and the host’s self-tissues. Thus, the detection of IgG or IgA antibodies against specific lectins may help to identify the offending foods so that they can be removed from the patient’s diet, thereby removing the antigenic stimulus and relieving the symptoms of the disease [26].

To show this cross-reactivity between different lectins and various tissue components, we reacted lectin-specific antibodies with 62 different tissue antigens [27]. The most reactive of the food antibodies was WGA, with 37 out of the 62 tissue antigens, followed by red kidney bean antibody with 20 out of 62, soybean antibody also with 20, then peanut agglutinin antibody with 15. Our results confirmed that anti-lectin antibodies do indeed bind with human tissues. We then sought to determine the prevalence of these antibodies in the blood of 500 nominally healthy donors. The percentage elevation of antibodies against different lectins were in the ranges of 12–16% for IgG, 9.7–14.7% for IgA, 12–18% for IgM, and 7.8–14.6% for IgE.

Finally, we then tested the lectin-specific antibody levels in sera that were positive for both RF and ANA in comparison to controls. The results showed that IgM anti-lectin antibody levels were highly correlated with RF levels, but not with ANA. This reaction of antilectin antibodies with human tissue antigens and their correlation with RF levels may indicate mechanisms by which antibodies produced against undigested lectins may be involved in the pathogenesis of autoimmune diseases such as RA [27].

**Figure 17:**
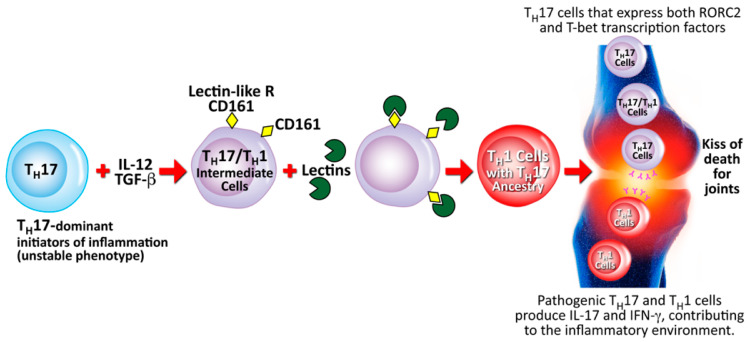
Proposed mechanism for the contribution of lectins to the conversion of Th17 to Th17/Th1 and towards rheumatoid arthritis. T_H_ = T-helper; IL = interleukin; TGF-β = transforming growth factor beta; CD = cluster of differentiation; ROR = retinoic acid-related orphan receptor; T-bet = T-box expressed in T cells; IFN-γ = interferon gamma.

Lectins may also induce autoimmunity through a different mechanism that involves cell-mediated immunity. This particular pathophysiological mechanism involves Th17 and Th1 cells from humans with autoimmune arthritis driven by CD161, which is a lectin-like receptor found on the surface of those cells [192]. Th17 cells are a subset of CD4^+^ cells that have shown proinflammatory activities in different autoimmune diseases, including autoimmune arthritis and collagen-induced arthritis [193,194,195]. Human Th17 cells are characterized by the CD161 lectin-like receptor and the production of the cytokine IL-17. In patients with arthritis, a cytokine phenotype that is unique to both Th1 and Th17 cells is expressed by the majority of IL-17-secreting cells within the joints. Within these inflamed joints, it has also been shown that both Th1- and Th17-specific transcription factors are expressed on the Th17/Th1 intermediate cells. It is possible that the binding of lectins to the lectin-like CD161 receptor on the Th17/Th1 intermediate cells is part of the mechanism for converting these cells to the Th1 and Th17 phenotypes that cause the inflammation in the joints. This mechanism is shown in Figure 17.

Based on the mechanism described above for the induction by lectins of autoimmunities, the authors propose that the elimination of certain dietary elements (such as lectins) that have a detrimental effect on both enterocyte and lymphocyte structure and function can reduce the antigenic stimulus, thereby reducing or possibly even eliminating the disease symptoms in some patients with autoimmune disorders triggered or associated with dietary factors.

## 6. A Brief Look at Infections, Autoimmune Diseases, and the Hygiene Hypothesis

There is such a wealth of material on the role of infections in the exposome and autoimmunity that we have relegated it to a second follow-up article. However, we can say that infections are a major part of the exposome and a major well-established contributor to autoimmune diseases. The hygiene hypothesis, which is both well-known and widely debated, bears mentioning and studying for the many useful insights it brings to light about infections and autoimmunity. It basically states that although infectious agents are potentially responsible for many diseases, both infectious and noninfectious, they could also have a favorable effect on such illnesses. The classic example is a child growing up on a farm versus a child growing up in the city. The farm child may run barefoot, encounter many children in germ-ridden environments, come in contact with animals, pick up parasites, and thus contract many diseases. The hygiene hypothesis proposes that the farm child will consequently build up immunities to all the diseases contracted and will become an adult with a stronger, more robust immune system. On the other hand, the city child will be protected from germs, perhaps excessively so, practicing the latest hygienic protocols, using the latest hygienic products and devices, encountering fewer children in relatively more germ-free environments, and have limited contact with animals. The city child will be protected from germs, parasites, and disease, but as a result will grow up to have an undeveloped immune system and be more susceptible to infections and other diseases. The classic hypothesis states that the reason for this is that humans are born with an immature immune system and an unhealthy Th2-biased immunotype, and that the proper stimuli, such as exposure to infections, will bring the child’s immune system to a healthy Th1/Th2 balance [30]. However, the classic hygiene hypothesis has been assailed through three decades with what appear to be contradictions or exceptions. This is because the expansion of scientific knowledge and the ever-continuing development of new technologies affect our perceptions of the mechanisms behind the cellular and humoral responses of the immune system. High-resolution flow cytometry and cell sorting now make it possible to determine the phenotypic characterization, function, and development of diverse classes, using monoclonal antibodies and the direct staining and counting of cells instead of cytokines. Thus, the originally proposed simplistic model of Th1 and Th2 immune responses was shaken up and expanded by the discovery that T lymphocytes were actually composed of a more complex immune network of subsets, going beyond Th1 and Th2 and including cell types such as CD4, CD8, Th17, Tregs, NK cells, and NKT cells [196,197,198,199,200,201]. As already shown above, the old concept of hygiene was somewhat individualistic, comparing one person going barefoot to another going shod, to whether one washed one’s hands or not. Most of the factors considered for the hygiene hypothesis today are collective, not individual, such as the quality of the environment’s drinking water, the preservation of food, and the extent of use of antibiotics and vaccines [197]. When Strachan originally proposed the hygiene hypothesis in 1989 [202], he proposed that common childhood infections may reduce the frequency of atopic diseases. Soon after, in 2000, he proposed that the increase in frequency of allergic disease in the past four decades could be ascribed to the decrease in infectious diseases [203]. In the early 2000s, the hygiene hypothesis was extended to autoimmune diseases [204]. At that time, data in experimental models already showed that infections, particularly parasitic infections, could prevent autoimmunity [205]. The investigation has spread to determining the respective roles of parasites, bacteria, viruses, pathogens, and commensal bacteria in immune response, allergies, autoimmunity, and the hygiene hypothesis itself [197]. In an apparent vindication of Strachan, a negative correlation has been observed between the decrease in the frequency of infectious diseases and the increase in allergic and autoimmune diseases [197]. One proposed mechanism for this is the phenomenon is known as antigenic competition, which could be applied to the hygiene hypothesis by supposing that very strong immune responses against infectious agents could result in weak responses against allergens or autoantigens due to increased consumption of homeostatic factors [196]. Another potential mechanism is epigenetic modification, a biochemical change in the chromatin that is functionally relevant, but does not affect the nucleotide sequence of the genome; these modifications play a key role in the differentiation of T-cell lineages and the balance between Th subsets, and classic examples of them are DNA methylation and histone modification. Intestinal commensals or microbiota have also been proposed to have a role in the hygiene hypothesis; understanding how microbiota impact susceptibility to and severity of infections could guide the development of therapies that can shift the host–pathogen–microbiota balance back to a healthier state [206]. In a recent editorial in a special gut microbiome issue of *The Journal of Immunology* published in 2021, Nagler summarized the relationship between the hypothesis and microbiota, saying that “lifestyle changes associated with industrialization, including increased sanitation, antibiotic use, consumption of processed foods, and urban living, have reduced microbial diversity and altered both community structure and function” [207]. There are many other mechanisms behind the new hygiene hypothesis that involve cytokines, toll-like receptors and ligands, and more. The hygiene hypothesis has a lot to teach us about immunity, immunopathology, infectious diseases, and autoimmunity, and the roles of lymphocyte subsets in their mechanisms.

## 7. Conclusions

Researchers and clinicians should put major emphasis on finding the root causes of pathophysiological changes that can occur years before the full onset of autoimmune diseases. These changes can be caused by both genetic and/or environmental factors, or by the genome and exposome. The exposome is an individual’s lifetime exposure to external and internal environmentally related factors. Three of the most important of these are toxic chemicals, food, and pathogens, all of which play significant roles in the pathophysiology of autoimmune diseases. A better understanding of these three environmental factors would undoubtedly facilitate the development of better treatment and disease management protocols for patients suffering from the more than 100 autoimmune diseases that affect a significant portion of the world population [14]. This article only focused on toxic chemicals and food, and we intend to devote a subsequent article to the role of infectious pathogens.

In relation to chemicals and their role in autoimmune diseases, we focused mainly on mercury and food coloring as contributors to the induction of ADs. Similar mechanisms may be applied to pesticides, herbicides, solvents, other heavy metals, plasticizers, preservatives, emulsifiers, nanoparticles, flame retardants, household cleaners, drugs, silica, silicone, exogenous sex hormones, cosmetics, hair dyes, acrylamide, glyphosates, and many more. Each deserves similar attention and clinical studies on their involvement in ADs.

Similarly, this review also examined foods such as wheat, milk, aquaporins, lectins, and agglutinins. We showed that molecular mimicry or peptide sequence homology is a common mechanism for immune reactivity and ADs, as we have shown chain correspondence between wheat components and neuronal antigens, between milk proteins and islet cells, and between plant aquaporins and human aquaporins [23,24,25,26,27,28,30,32,126,152,173]. We showed that 86 out of 180 foods cross-reacted with human α-synuclein [187], and that more than 50 out of 204 tested food antigens cross-reacted with thyroid target sites and tissues associated with type 1 diabetes [208,209]. We have demonstrated that lectins can not only lead to ADs through molecular mimicry, but also through lectin-like receptors on self-tissues [190,191]. Such significant structural similarities can lead first to food immune reactivity, which can then lead to the immune system attacking the body’s own tissues and full-blown autoimmune diseases such as MS, NMO, and other neuroimmune disorders. In fact, one of our studies published in 2018 [29] found that the reactivity between numerous food antigens and neuronal amyloid-beta-peptide-42 may play a crucial role in Alzheimer’s disease. Many other foods should also be investigated for their pathophysiological roles in ADs.

Based on the information above, as we stated at the beginning of these concluding remarks, we feel it is paramount for researchers and practitioners to detect or identify the exact causes of the pathophysiological changes or symptomatology of individuals potentially or suspected of suffering from ADs. Once the actual triggers are identified, they can be removed, and treatment protocols can be devised that include the repair of their damaged immune systems. This strategy of DETECT–REMOVE–REPAIR is one that should be applied to all autoimmune diseases.

The role of pathogens in the pathophysiology of autoimmune diseases will be further reviewed in a proposed Part II of this article

To better illustrate the role of the exposome and its factors (such as toxic chemicals and dietary proteins) in different autoimmune diseases, a summary table with pertinent references is provided below (Table 1).

### 7.1. Strengths

A strength of our review was the inclusion of extensive studies, including those performed in our own laboratory, in relation to the role of toxic chemicals, food proteins, and peptides in the pathophysiology of autoimmune disease that affect a significant portion of the world population.

Another strength was the presentation of the mechanisms behind the chemical induction of neoantigen formation and molecular mimicry between dietary proteins/peptides and various human autoantigens. Understanding these mechanisms would facilitate the development of better treatment strategies, particularly the removal of these factors from the lifestyles of patients with various autoimmune diseases.

### 7.2. Limitations

While the focus of this review was undoubtedly on two major exposome factors, toxic chemicals and food, the exposome is an individual’s exposure to a variety of external and internal factors (as shown in Figure 1), not all of which were covered in this article. Even in discussing the role of toxic chemicals in autoimmune diseases, we chose mercury and food coloring as only two of the more than 100,000 new chemicals that have been introduced into the industrialized world since the 1940s. Each and every one of those chemicals deserves similar attention for their possible role in autoimmune diseases. Similarly, out of thousands of food proteins and peptides, we discussed only the role of wheat, milk, aquaporins, lectins, and agglutinins in the pathophysiology of autoimmune diseases. In our earlier studies, we showed that 86 out of 180 foods cross-reacted with human α-synuclein, and more than 50 out of 204 tested food antigens cross-reacted with thyroid antigen and tissue associated with type 1 diabetes [190,208,209]. The extensive cross-reactivity that we did demonstrate between food proteins/peptides and human tissue antigens that we selected may encourage further research about the thousands of other food antigens that may play a role in the development of autoimmune disease.

## Figures and Tables

**Table 1:** Summary of environmental factors, associated diseases, and corresponding references.

Environmental Factor	Associated Disease	Reference and Number
Crystalline silica	RA, SLE, SSc, UC	Pollard et al. [55]
Smoking	RA, SLE, MS, TA, IBD	Pollard et al. [56]
Solvents	SSc, MS	Miller et al. [57]
Cosmetics	RA, SLE, PBC	Pollard et al. [58]
Mercury	TA	Gallagher et al. [70]
		Somers et al. [71]
Mercury	Nephrotic syndrome	Miller et al. [72]
Food coloring	Rising incidence of autoimmune	Lerner and Matthias [33]
	disease	
Food coloring	ADD	Carter [95]
	ADHD	Boris and Mandel [96]
	Hypersensitivity	Weliky and Heiner [103]
Wheat gluten and nongluten	CD	Arentz-Hansen et al. [120]
proteins and peptides	NCGS, Crohn’s disease	Vojdani [126]
	Autoimmune thyroid disease	Counsell et al. [129]
	Osteoporosis	Sugai et al. [131]
	Autoimmune myocarditis	Frustaci et al. [132]
	Dermatitis herpetiformis	Sárdy et al. [133]
	Gluten ataxia	Hadjivassiliou et al. [135]
	Choreic syndrome	Pereira et al. [137]
	MS	Shor et al. [141]
	NMO	Jacob et al. [142]
	Alzheimer’s disease	Vojdani [28]
		Vojdani and Vojdani [29]
Milk, caseins, alpha and beta	Type 1 diabetes	Virtanen et al. [149]
lactalbumin	EAE	Stefferl et al. [151]
	Autism	Vojdani et al. [152]
	MS	Guggenmos et al. [153]
	SLE	Riemekasten et al. [154]
	Uveitis	Wildner and Diedrichs-
		Môhring
	Alzheimer’s disease	Vojdani [28]
		Vojdani and Vojdani [29]
Aquaporins from human,	NMO	Jarius and Wildemann [170]
tomato, corn, soy, spinach		Vaishnav et al. [171]
	MS	Vojdani et al. [174]
α-synuclein-containing food	PD	Hawkes et al. [187] Vojdani et al. [189]
Lectins and agglutinins	Autoimmune diseases	Vojdani [26]
		Vojdani et al. [27]

RA = rheumatoid arthritis; SLE = systemic lupus erythematosus; SSc = systemic sclerosis; UC = ulcerative colitis; MS = multiple sclerosis; TA = thyroid autoimmunity; IBD = inflammatory bowel disease; PBC = primary biliary cholangitis; ADD = attention deficit disorder; ADHD = attention-deficit/hyperactivity disorder; CD = celiac disease; NCGS = nonceliac gluten sensitivity; NMO = neuromyelitis optica; EAE = experimental autoimmune encephalomyelitis; PD = Parkinson’s disease.

## Data Availability

Not applicable.
